# Torquay a Resort for Consumptives

**Published:** 1857-07

**Authors:** 


### TORQUAY, A RESORT FOR CONSUMPTIVES.†

In this work one of our most accomplished provincial phy-

---

† Torquay in its Medical Aspect as a Resort for Pulmonary Invalids. By C. Radclyffe Hall, M.D. London: Churchill. Torquay: Cockrem. 1857.

sicians supports the view of the rational cure of consumption by attention mainly to the laws of life. "In the machinery of animal life," says this author, with great force, "the main condition of strength is *exercise within the limits of power.*" Torquay, according to Dr. Hall, presents special advantages, in that it allows the consumptive invalid to take outdoor exercise very constantly throughout the winter. And the winter here is short; it arrives so late, and spring so early, as constantly to excite remark amongst the natives from the north who visit this part of the south. Dr. Hall recommends that a consumptive invalid should not remain the whole year at Torquay. It is a double advantage for him to leave for June, July and August. In the seventh chapter, the author supplies, from Mr. Vivian, the following meteorological facts:—

"The mean *annual* temperature of Torquay was (omitting decimals) two degrees (F.) higher than that of all England. The maximum temperature for the whole year was seven degrees lower. The minimum temperature was twelve degrees higher. The mean daily range of temperature was five degrees less. The mean quarterly range of temperature was thirty-one degrees less. The average number of days on which the rain fell was fifteen days less. The depth of rain was two inches greater. The average quantity of vapour contained in the air for the whole year was the same. The additional quantity of watery vapour required to saturate a cubic foot of air was greater at Torquay by ·2 (Torquay, ·9; allEngland, ·7). The average humidity of Torquay was ·6 less (Torquay ·76; all England, ·82)."

Dr. Hall's book well deserves the careful study of all who wish to find an Atlantis for consumptive patients or friends.

